# The Canonisation of Research

**Published:** 1916-03-11

**Authors:** 


### THE CANONISATION OF RESEARCH.

To judge from various public expressions of opinion there seems to be a widespread view that as a nation we have suffered both in the economic struggle and in the present war because neither our teaching organisations nor our manufacturers have regarded the art of research at its true value. Corresponding to this there appear in the public prints protests from heads of academic bodies to the effect that they at least have been researchers or that they will become so in the future. Even the most ancient of our Universities publicly dons the white sheet of the penitent and announces its intention to reform, while from Bristol comes the assurance that the position of research, in this recently arrived member of the academic family, "has indeed been recognised to a somewhat unusual degree." These assurances have their amusing side and tell us what perhaps we had heard before, that "Codlin is the friend—not Short." But they carry also the intimation that war, in spite of its penalties, has some compensations, and that one of these is a stirring among bones which had previously been regarded as "dry" or even as dead. When a stimulus has actually produced a response from the "home of lost causes," it can hardly be doubted that other activities have felt its force.

In these columns there is certainly no desire to say anything which would seem to undervalue attempts to penetrate the still unlocked secrets of Nature or to establish new scientific truths. And we quite agree that one of the functions of a University is to cultivate this aim and to provide means for its attainment. A University professor, most certainly, may be judged, at least in part, by the ambitions which he awakes in his pupils, and by the work produced under his inspiration. At the same time we think there is a possibility that this new cry may lead to a wrong standard of judgment. It is a pleasing picture, that of a number of young and ardent spirits crowding the laboratory benches and each set on a research which will write for him a local fame and will incidentally do something perhaps for "the relief of man's estate." But is such an arrangement in practice at all likely to attain to much real value? Indeed, by an overstatement of the case positive harm may be done, for it may fashion in the academic atmosphere a totally false criterion. Research has a dignified and an adventurous quality which may well possess the soul to the prejudice of such homely attainments as the mere equipment and disciplining of the mind with already known truths. Yet for the vast majority this latter is the true ambition. In a word, we should hold that research to be of any value is for the selected few, and that one of the responsibilities of a University is to discover these and to give them encouragement and opportunity. But to attempt to turn the main stream of academic work in the direction of something which can be called research is likely rather to manufacture prigs than to advance knowledge.

That Universities and kindred institutions have hitherto fallen short in their responsibilities in reference to research work is doubtless true, and even more emphatically true is it that manufacturers have failed to appreciate the important bearings of scientific investigations on industrial advance. These propositions have been advanced again and again, and the war has lent to them a new emphasis. But, important as they are, they must be stated in due proportion. Experience will doubtless secure this, and the fond anticipation that every student, if only rightly guided, will achieve success as a "researcher" is doomed to disappointment.

